# Rising incidence of HPV positive oropharyngeal cancer in Taiwan between 1999 and 2014 where betel nut chewing is common

**DOI:** 10.1186/s12885-022-09407-5

**Published:** 2022-03-21

**Authors:** Cheng-Ping Wang, Tseng-Cheng Chen, Wan-Lun Hsu, Jenn-Ren Hsiao, Peir-Rong Chen, Mu-Kuan Chen, Chun-Hung Hua, Ming-Hsui Tsai, Jenq-Yuh Ko, Pei-Jen Lou, Chun-Ju Chiang, Chen-Tu Wu, Yih-Leong Chang

**Affiliations:** 1 Department of Otolaryngology, College of Medicine, National Taiwan University Hospital and National Taiwan University, 7 Chung-Shan South Rd., Taipei, Taiwan; 2 Genomics Research Center, Academia Sinica, Taipei, Taiwan; 3 Department of Otolaryngology, College of Medicine, National Cheng Kung University Hospital, National Cheng Kung University, Tainan, Taiwan; 4 Department of Otolaryngology, Hualien Tzu Chi Hospital and Tzu Chi University, Hualien, Taiwan; 5 Department of Otorhinolaryngology, Head and Neck Surgery, Changhua Christian Hospital, Changhua, Taiwan; 6 Department of Otorhinolaryngology, China Medical University Hospital, Taichung, Taiwan; 7 Graduate Institute of Epidemiology and Preventive Medicine, College of Public Health, National Taiwan University, Taipei, Taiwan; 8 Department of Pathology, National Taiwan University Hospital and National Taiwan University College of Medicine, Taipei, Taiwan

**Keywords:** Oropharyngeal cancer, Human papillomavirus, p16, Betel nut, Incidence

## Abstract

**Background:**

The incidence of human papillomavirus (HPV) positive oropharyngeal cancer (OPC) is rising but HPV negative OPC is decreasing in Western countries. In Taiwan, the incidence of HPV negative OPC is common but the incidence of HPV positive OPC remains unknown. The objective of this study is to estimate the incidence trend and the survival of HPV positive OPC in Taiwan.

**Methods:**

Between 1999 and 2014, primary tumor tissues from 425 incident OPCs were obtained from 5 medical centers in Taiwan. 408 OPCs were evaluated by the EasyChip HPV genotyping (King-Car, I-Lan, Taiwan) and 369 OPCs by p16 staining. The clinical data were retrospectively obtained from the medical records.

**Results:**

In our study, 29% of OPCs were HPV positive. The percentage of HPV positive OPC was stable from 1999 to 2014 (25% (1999–2002), 30% (2003–2006), 30% (2007–2010), 29% (2011–2014)). The estimated crude incidence rate of HPV positive OPC increased significantly from 0.62 (1999–2002), 1.06 (2003–2006), 1.52 (2007–2010) to 1.74 (2011–2014) per 100,000 person-year. The sensitivity and specificity of p16 staining for positive HPV infection were 92% and 91%, respectively. The 5-year overall survival rates for patients with HPV positive OPC and with HPV negative OPC were 67.8% and 49.0%, respectively (HR = 0.52 (0.35–0.76), *p* = 0.0005). Patients with HPV positive OPC but no betel nut/cigarette exposure had the best overall survival (5-year: 88.2%, *p* < 0.0001). Patients with HPV negative OPC and betel nut/cigarette exposure had the worst overall survival (5-year: 46.6%, *p* < 0.0001). Patients with HPV positive OPC but also with betel nut/cigarette exposure had poorer 5-year overall survival (48.3%, *p* < 0.01).

**Conclusion:**

The incidence of HPV positive OPC is increasing along with HPV negative OPC, which leads to stably low percentage of HPV positive OPC in Taiwan. HPV positive OPC may become an important head and neck cancer when the incidence of HPV negative OPC declines in the near future. P16 is a useful surrogate marker for HPV infection in OPC and a good prognostic indicator for treatment outcome of OPC. Patients with HPV positive OPC but no betel nut/cigarette exposure has an excellent prognosis. Betel nut/cigarette exposure significantly worsens the prognosis of HPV positive OPC.

## Introduction

In recent decades, human papillomavirus (HPV) positive oropharyngeal squamous cell carcinoma (OPC) is clearly becoming one of the most common head and neck cancers in developed countries in Northern America, Europe, Australia and Japan [1–5]. Sexual behavioral changes have a strong association with rapidly increasing incidence of HPV positive OPC [1–5]. On the contrary, the incidences of other cigarette-associated head and neck cancers including HPV negative OPC are significantly decreasing because of reduced cigarette consumption. Therefore, HPV positive OPC is increasing in the proportion of overall OPC and more important than HPV negative OPC in those countries [1–5]. In addition, the mutation landscape of HPV positive OPC is significantly different from that of HPV negative OPC and HPV positive OPC is very sensitive to chemoradiation with much better prognosis than HPV negative OPC. Together, HPV positive OPC is a truly unique head and neck cancer and different treatment approaches have been under investigation to reduce treatment side effects [6, 7].

In many countries, the percentage of HPV positive OPC remains low and therefore, clinicians still treat mostly HPV negative OPC. The low and stable incidence of HPV positive OPC may be one of the reasons. However, another possibility is that the increasing incidence of HPV positive OPC is obscured by persistently increasing incidence of HPV negative OPC.

In Taiwan, the percentage of HPV positive OPC remained low around 17% -30% [8–12]. As a result, most clinicians pay more attention to HPV negative OPC without realizing the importance of HPV positive OPC in clinical practice nor in public health [1, 13–15]. Furthermore, some clinicians questioned the role of p16 and HPV in prediction of OPC prognosis because most OPC in Taiwan are associated to exposure of traditional risk factors including alcohol, betel nut and cigarette (ABC); this is true even for HPV positive OPC. However, it may be possible that HPV positive OPC is becoming one of the most important head and neck cancers, and p16 or the presence of HPV in tumor is a surrogate predictive indicator for prognosis of OPC in Taiwan.

In this study, we estimate the incidence trend of HPV positive OPC by genotyping HPV DNA from OPC tumor tissues collected at diagnosis from 5 medical centers located in different regions of Taiwan to extrapolate the future trend of OPC in Taiwan. We also evaluate and discuss the role and survival impact of HPV infection and p16 in OPC patients in Taiwan. Our findings may help to inform other countries where the prevalence of HPV positive OPC remains stably low and ABC-associated head and neck cancers are still prevalent.

## Patients and methods

### Patients

The study included 425 patients with newly diagnosed oropharyngeal cancer without previous or treated head and neck cancer between 1999 and 2014 from five medical centers (265 patients from National Taiwan University Hospital (NTUH) in Northern Taiwan, 19 from China Medical University Hospital (CMUH) and 38 Changhua Christian Hospital (CCH) in Central Taiwan, 59 from National Cheng-Kong University Hospital (NCKUH) in Southern Taiwan and 44 from HuaLien Tzu Chi Hospital (HTCH) in Eastern Taiwan), all of whom participated in the Taiwan Biosignature Project (Taiwan Head and Neck Cancer Study Group). The ratio of male to female patients was 9.3, which was similar to the nationwide data during the same time period [16]. The age distribution and primary tumor location in this study were also similar to nationwide data [16]. Formalin-fixed, paraffin-embedded tumor tissues were all collected from primary tumor of OPC before treatment and pathologically re-confirmed squamous cell carcinoma by two head and neck pathologists (Y-L.C. and C-T.W.) at NTUH at the time of the study. Recurrent or metastastic tumor were excluded. All tumors were processed for HPV PCR genotyping analysis and p16 immunohistochemical staining at NTUH. Patients’ data including age at diagnosis, sex, risk factor exposure about betel nut chewing, cigarette smoking and alcohol drinking, AJCC TNM stage and treatment were obtained through chart review. Survival data were obtained from medical chart review and National Death Registry. This study was reviewed and approved by the institutional review boards of all five hospitals including NTUH, CMUH, CCH, NCKUH and HTCH, and Academia Sinica.

### Laboratory methods

#### HPV PCR analysis

We used a commercial EasyChip® HPV blot kit (King Car, Taiwan) to carry out HPV genotyping of polymerase chain reaction (PCR) of HPV and GAPDH. The quality of the HPV blot met the requirement for class III GMP certification. The kit allowed specific detection of 39 HPV genotypes, including low risk, probable risk and high risk types (HPV types 6, 11, 16, 18, 26, 31, 32, 33, 35, 37, 39, 42, 43, 44, 45, 51, 52, 53, 54, 55, 56, 58, 59, 61, 62, 66, 67, 68, 69, 70, 72, 74, 82, CP8061, CP8304, L1AE5, MM4, MM7 and MM8, as well as three intrinsic controls) and is based on reverse hybridization. Detailed procedures of HPV genotype determination are described in previous literature [17, 18].

#### p16 immunohistochemical (IHC) staining

4-μm thickness sections from primary tumor were deparaffinized and pre-treated for antigen retrieval by autoclave heating (121 °C) in 10 mM sodium citrate buffer (pH 6.0) for 10 min [19]. These sections were blocked for endogenous peroxidase activity with 3% H2O2 in methanol for 10 min and then washed in phosphate-buffered saline (PBS). Thereafter, the sections were immersed in UltraVision Protein Block (Thermo Fisher Scientific, Fremont, LA, USA) for 10 min, covered with a primary rabbit monoclonal antibody specific for p16 (clone: EP1215Y, Epitomics, Abcam Company, Burlingame, CA, USA) and incubated for one hour at room temperature. Immunoreactions were performed using UltraVision Quanto Detection System HRP DAB (Thermo Fisher Scientific, Fremont, LA, USA). Immunohistochemical evaluation of p16 in OPSCC specimens was based on the intensity and extent of nuclear and cytoplasmic reactivity. Positive p16 expression was defined as strong and diffuse nuclear and cytoplasmic staining in 70% or more of the tumor cells [7]. Two independent pathologists (Y-L.C. and C-T.W.) were involved in the assessment of tumor p16 expression.

## Statistics

We presented the overall percentage of HPV positive OPC classified by HPV PCR test and p16 IHC staining by calendar year and by four different regions of Taiwan. Chi-square tests was used to determine the difference in age, gender, cigarette smoking, alcohol drinking, betel quid chewing, clinical stage and tumor location between HPV positive and HPV negative OPCs. Patients were stratified by calendar year (four-year categories: 1999–2002, 2003–2006, 2007–2010 and 2011–2014) and incidence rate was calculated for HPV-positive OPC using the percentage of HPV positive OPC and the average crude incidence rate of OPC obtained from Taiwan Cancer Registry using the third version of head and neck surgery and oncology definition for each calendar year group [16]. Age-adjusted incidence rate was also estimated. Follow-up ended at either death or 2015 and the overall survival rate was estimated using Kaplan–Meier method, comparing the groups using log-rank test. Survival analysis was calculated by HPV PCR and p16 IHC staining results, with/without betel nut chewing and/or cigarette smoking. All statistical analyses were performed using the SPSS software package, version 16.0 (SPSS Inc., Chicago, IL). A *p*-value < 0.05 was considered statistically significant.

## Results

### The percentage and incidence trend of HPV positive OPC estimated by HPV PCR analysis

**Fig. 1:**
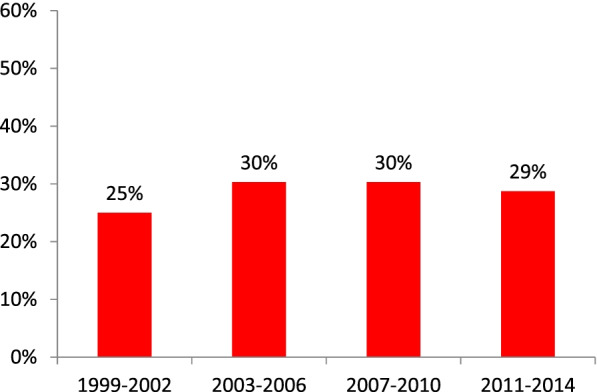
Prevalence of HPV positive OPC in 408 OPCs across four calendar periods (1999 to 2002, 2003 to 2006, 2007 to 2010, and 2010 to 2014) determined by HPV PCR

**Fig. 2:**
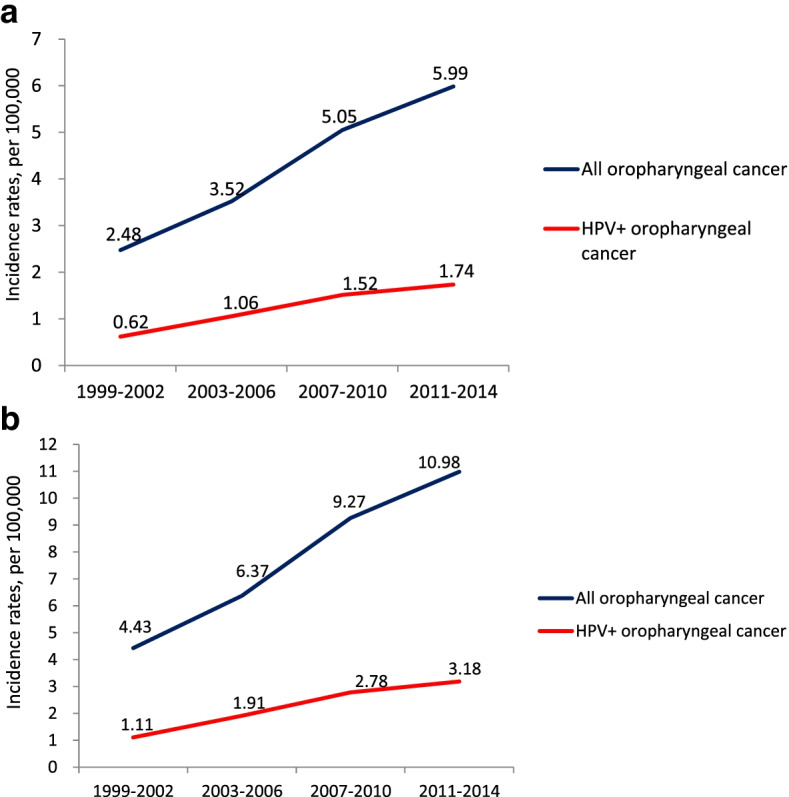
**a** Estimated crude incidence rates of HPV positive OPC across four calendar periods (1999 to 2002, 2003 to 2006, 2007 to 2010, and 2010 to 2014) determined by HPV PCR. Blue line represents the average crude incidence rate of all OPC including man and woman during the 4 calendar years in Taiwan. Red line represents the estimated average crude incidence rate of HPV positive OPC including man and woman during the 4 calendar years in Taiwan. **b** Estimated crude incidence rates of male HPV positive OPC across four calendar periods (1999 to 2002, 2003 to 2006, 2007 to 2010, and 2010 to 2014) determined by HPV PCR. The blue line represents the average crude incidence rate of all male OPC during the 4 calendar years in Taiwan. The red line represents the estimated average crude incidence rate of male HPV positive OPC during the 4 calendar years in Taiwan

Of the 425 OPC tumors, HPV PCR test was successfully performed on 408 (96%) samples. 119 (29%) of OPC tumors were HPV positive, including 83 out of 248 tumors (33%) in NTUH (Northern Taiwan), 13 out of 57 tumors (23%) in CMUH and CCH (Central Taiwan), 15 out of 59 tumors (25%) in NCKUH (Southern Taiwan) and 8 out of 44 (18%) in HTCH (Eastern Taiwan). By calendar year groups, the percentage of HPV positive OPC was 25% (1999–2002), 30% (2003–2006), 30% (2007–2010) and 29% (2011–2014), which showed stability across calendar periods (Fig. 1). The estimated crude incidence rate of HPV positive OPC was 0.62 (1999–2002), 1.06 (2003–2006), 1.52 (2007–2010) and 1.74 (2011–2014) per 100,000 person-year, which significantly increased across calendar periods by 181% between the period of 1999 to 2002 and the period of 2011 to 2014 (Fig. 2a). The estimated crude incidence rate of male HPV positive OPC was 1.11 (1999–2002), 1.91 (2003–2006), 2.78 (2007–2010) and 3.18 (2011–2014) per 100,000 person-year, which also significantly increased across calendar periods by 186% from the period of 1999 to 2002 to the period of 2011 to 2014 (Fig. 2b). The estimated age-adjusted incidence rate of HPV positive OPC was 0.60 (1999–2002), 0.92 (2003–2006), 1.20 (2007–2010) and 1.27 (2011–2014) per 100,000 person-year, which significantly increased across calendar periods by 112% from the period of 1999 to 2002 to the period of 2011 to 2014. During the same period of time, the crude incidence rate of oral cancer was 11.35 (1999–2002), 14.93 (2003–2006), 18.23 (2007–2010) and 20.82 (2011–2014) per 100,000 person-year, which also significantly increased across calendar periods by 83% from the period of 1999 to 2002 to the period of 2011 to 2014.

**Fig. 3:**
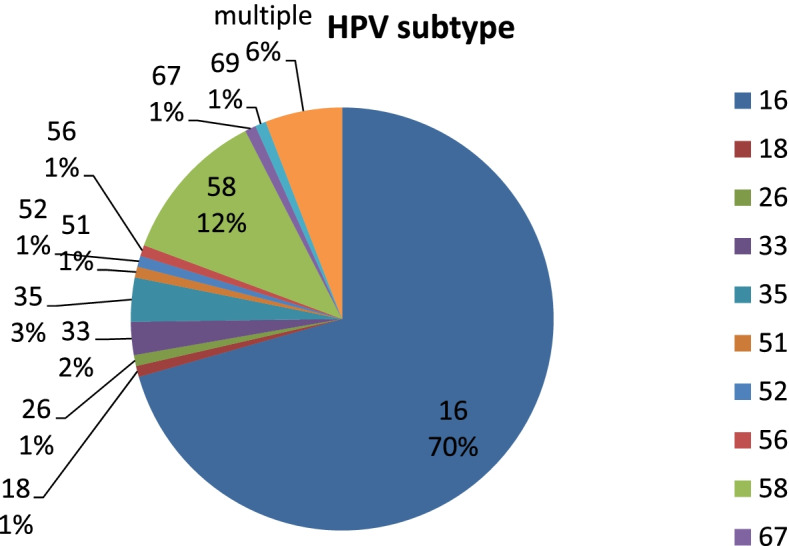
HPV subtype distribution of 119 HPV positive OPCs by HPV PCR genotyping

Among 119 HPV positive OPC, HPV subtype distribution was 70% for HPV16 (*n* = 84), 12% for HPV58 (*n* = 14), 3% HPV35 (*n* = 4), 2% for HPV33 (*n* = 3), 1% for HPV18 (*n* = 1),1% for HPV26 (*n* = 1), 1% for HPV51 (*n* = 1), 1% for HPV52 (*n* = 1), 1% for HPV56 (*n* = 1), 1% for HPV67 (*n* = 1), 1% for HPV69 (*n* = 1) and 6% for multiple infection (*n* = 7, including 6,16,18, 42, 51, 53, 56, 58, 62) (Fig. 3).

### The percentage of positive p16 OPC and the correlation between p16 staining and HPV PCR

Of the 425 OPC tumors, 369 (87%) were successfully p16 IHC stained. The percentage of positive p16 OPC was 31% (114 tumors). By calendar year groups, the prevalence of positive p16 OPC was 24% (1999–2002), 33% (2003–2006), 34% (2007–2010) and 28% (2011–2014), which showed stability across calendar periods and was similar to the result of the HPV PCR test. Of all tumors, 350 tumors successfully underwent both HPV PCR testing and p16 IHC staining. Table 1 shows that the sensitivity and specificity of p16 IHC staining predicting for positive HPV infection in OPC were 92% and 91%, respectively. The positive predictive and negative predictive values were 80% and 97%, respectively (*p* < 0.0001).

**Table 1:** Correlation between HPV PCR and p16 IHC staining in OPC (350 patients with both tests) (*p* < .0001)

Tests		HPV PCR		
		Positive	Negative	Total
p16 IHC staining	Positive	88	22	110
	Negative	8	232	240
	Total	96	254	350

### The comparisons between clinical characteristics of OPC patients stratified by HPV PCR and p16 IHC staining

The comparisons between clinical characteristics of OPC patients stratified by HPV PCR and p16 IHC staining of OPC tumor are shown in Table 2. More than 70% of male OPCs were HPV negative, in contrast, about 60% of female OPCs were HPV positive (*p* < 0.01). HPV negative OPC patients were more likely to drink alcohol, chew betel nut or smoke cigarette. In contrast, more than half of HPV positive OPC patients, either defined by HPV PCR or p16 staining, had no exposure to ABC (*p* < 0.01). Two-thirds of OPC in patients without ABC exposure were HPV positive. HPV negative OPC was more locally advanced (T classification), whereas HPV positive OPC had more advanced nodal disease (*N* classification) (*p* < 0.01). About 40% of tonsillar cancers were HPV positive, whereas more than 80% of both tongue base and soft palate cancers were HPV negative (*p* < 0.01).

**Table 2:** The association between clinical characteristics of 425 OPC patients an HPV positive OPC defined by HPV PCR testing and p16 IHC staining

	**HPV PCR**			**p16 IHC**		
	Negative	Positive	*P* value	Negative	Positive	*P* value
Age (years)						
< 50	104 (72.7)	39 (27.3)	0.45	93 (71.5)	37 (28.5)	0.48
50–60	113 (72.9)	42 (27.1)		100 (69.9)	43 (30.1)	
> 60	71 (66.4)	36 (33.6)		61 (64.2)	34 (35.8)	
Gender						
Male	271 (74.3)	94 (25.8)	< 0.01	241 (71.4)	92 (27.6)	< 0.01
Female	17 (42.5)	23 (57.5)		13 (37.1)	22 (62.9)	
Cigarette smoking						
Never	44 (50.6)	43 (49.4)	< 0.01	36 (48.0)	39 (52.0)	< 0.01
Ever	199 (77.1)	9 (22.9)		175 (75.1)	58 (24.9)	
Alcohol drinking						
Never	57 (49.1)	59 (50.9)	< 0.01	51 (49.0)	53 (51.0)	< 0.01
Ever	162 (80.6)	39 (19.4)		145 (78.4)	40 (21.6)	
Betel quid chewing						
Never	92 (54.4)	77 (45.6)	< 0.01	78 (51.7)	73 (48.3)	< 0.01
Ever	128 (85.9)	21 (14.1)		119 (86.2)	19 (13.8)	
T classification						
T1	58 (20.0)	27 (23.0)	< 0.01	46 (18.1)	32 (28.0)	< 0.01
T2	82 (27.8)	50 (42.7)		73 (28.7)	39 (34.2)	
T3	37 (12.8)	17 (14.5)		33 (13.0)	20 (17.5)	
T4	76 (26.4)	13 (11.1)		68 (26.8)	13 (11.4)	
N classification						
N0	82 (27.8)	17 (14.5)	< 0.01	71 (28.0)	17 (14.9)	< 0.01
N1	33 (11.4)	9 (7.7)		26 (10.2)	10 (8.8)	
N2	111 (38.5)	72 (61.5)		98 (38.6)	65 (57.0)	
N3	27 (9.3)	9 (7.7)		25 (9.8)	12 (10.5)	
Overall stage						
I	32 (11.1)	4 (3.4)	< 0.01	25 (9.8)	6 (5.2)	0.13
II	28 (9.7)	9 (7.7)		25 (9.8)	8 (7.0)	
III	38 (13.1)	10 (8.5)		31 (12.2)	11 (9.6)	
IV	155 (53.8)	84 (71.8)		139 (54.7)	79 (69.3)	
Location						
Tonsil	131 (59.5)	89 (40.5)	< 0.01	115 (57.5)	85 (42.5)	< 0.01
Tongue base	69 (82.1)	15 (17.9)		64 (80.0)	16 (20.0)	
Soft palate	66 (91.7)	6 ( 8.3)		57 (90.5)	6 (9.5)	
Unspecific	22 (75.9)	7 (24.1)		18 (72.0)	7 (28.0)	

### The impacts of HPV PCR and p16 on overall survival of OPC patients

**Fig. 4:**
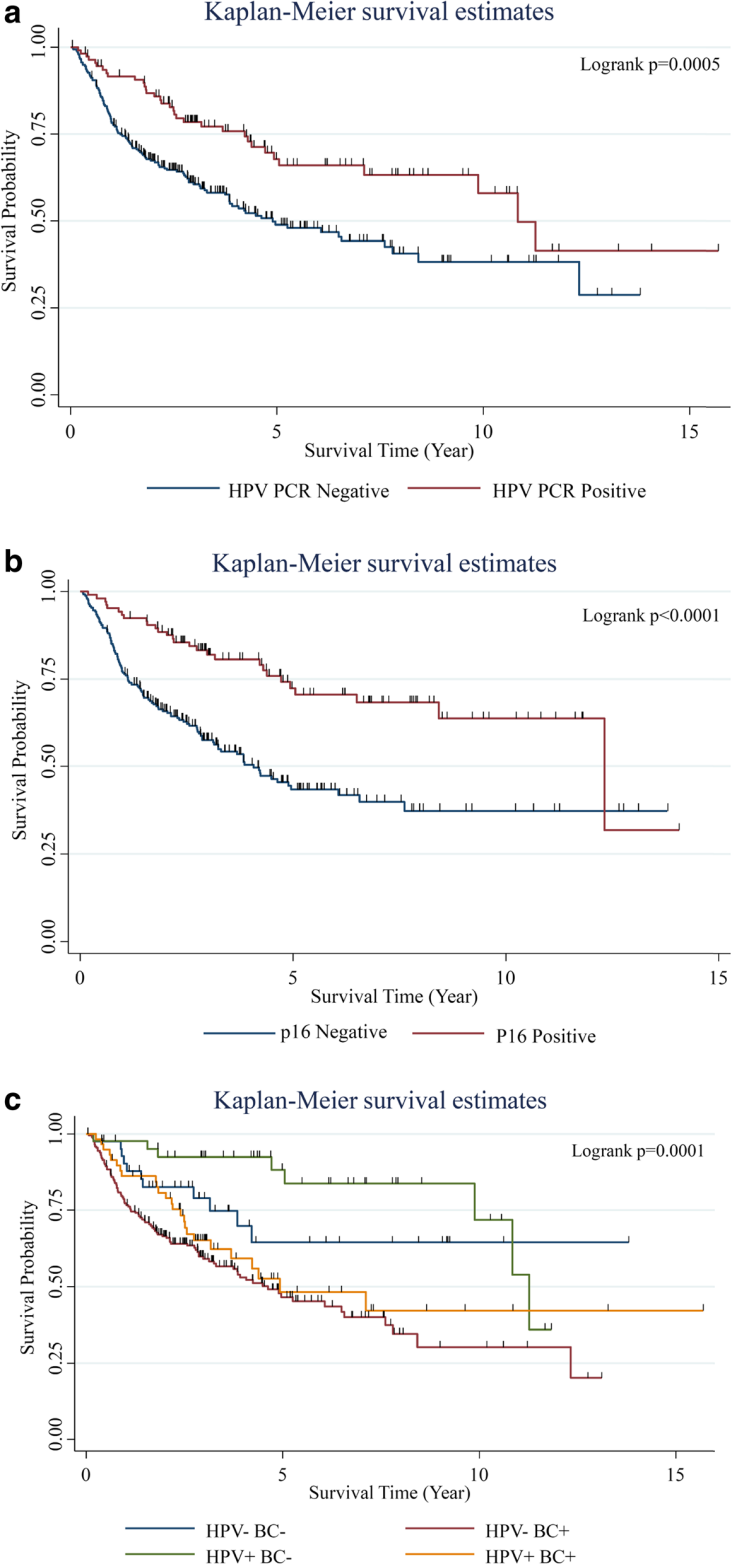
Overall survival curves of the OPC patients stratified by HPV PCR testing (**a**) and p16 IHC staining (**b**) in Taiwan. Impact of betel nut/cigarette (BC) exposure on the overall survival of HPV positive OPC patients (**c**)

The overall survival curves of OPC patients stratified by HPV PCR test and p16 IHC staining are shown in Fig. 4. The overall survival of patients with HPV positive OPC was significantly better than HPV negative OPC (HR: 0.52 (0.35–0.76), *p* = 0.0005). The 5-year overall survival rates of patients with HPV positive OPC and with HPV negative OPC were 67.8% and 49.0%, respectively. The overall survival of patients with p16 positive OPC was also significantly better than p16 negative OPC (HR: 0.39 (0.26–0.59), *p* < 0.0001). The 5-year overall survival rates of patients with p16 positive OPC and with p16 negative OPC were 72.4% and 43.4%, respectively. Patients with HPV positive OPC but no betel nut/cigarette exposure had the best overall survival (5-year rate:88.2%, *p* < 0.0001). Patients with HPV negative OPC and betel nut/cigarette exposure had the worst overall survival (5-year rate:46.6%, *p* < 0.0001). Patients with HPV positive OPC but also with betel nut/cigarette exposure had poorer overall survival (5-year rate: 48.3%, *p* < 0.01) but still tended to fair better than HPV negative OPC and betel nut/cigarette exposure (*p* = 0.087). Multivariate analysis showed that cigarette smoking (HR: 2.18 (1.05–4.55)), T classification (HR: 2.44 (1.31–4.57) for T4 tumor), N classification (HR:2.07 (1.06–4.02) for N2/3 tumor) and p16 (HR: 0.47 (0.28–0.78) for positive p16) were the independent factors for overall survival.

## Discussion

This is the first study on estimating the incidence trend of HPV positive OPC using primary tumor tissue obtained from multiple hospitals in Taiwan. Previous studies estimated incidence trend of HPV positive OPC using tumor tissue from a single hospital without time trend analysis [8–12] or nationwide data analysis without tumor tissue study [20]. This study also explores the incidence trend of HPV positive OPC in a population where HPV negative head and neck cancer is still prevalent. Similar to previous studies, this study shows that the percentage of HPV positive OPC remained stable around 30% for each calendar periods [8–12] but both incidences of HPV negative OPC and HPV positive OPC are rapidly increasing by about two folds in Taiwan during the period from 1999 to 2014. This low percentage may be one of the reasons why some clinicians do not think that HPV positive OPC is an important head and neck cancer in Taiwan despite the increasing number of HPV positive OPC patients in recent years.

In countries where the majority of OPC is HPV positive, the incidence of HPV positive OPC is rising but HPV negative OPC and other ABC-related head and neck cancers are declining or increasing slowly, resulting in the crossover of HPV positive OPC incidence and proportion with HPV negative OPC. In Taiwan, where betel nut chewing is common in the past years, peaking for males in 1997–2005 [14–16], the crude and age-adjusted incidence rates of oral cancer are still rising from 1999 to 2014 due to delayed carcinogen effect of betel nut [14, 15]. This implies that the incidence of ABC-related HPV negative OPC is also rising in a similar trend in the backdrop of a stably low but increasing HPV positive OPC incidence. The Taiwan Cancer Registry started collecting p16 status of OPC in 2018 and recently reported that only 25% of OPC (96.7% completion rate for p16 data among OPC) were p16 positive in 2018. The estimated crude incidence of HPV positive OPC in 2018 is 1.715 per 100,000 person-year, which is similar to our study. This may be because the crude incidence rate of oral cancer, the leading head and neck cancer, is still rising in 2018 with a similar rate as before in Taiwan [16].

Projections have reported that new case number and crude incidence rate of ABC-related oral cancer will plateau between 2020 and 2025 in Taiwan and then start to decline in the near future due to decreasing prevalence of betel nut chewing and cigarette smoking after peaking 20–30 years ago [14–16]. This implies that the incidence of HPV negative OPC will plateau and decline during the same period of time, too. On the contrary, new case number and crude incidence rate of HPV positive OPC will keep increasing because sexual behavioral changes and the pattern of oral HPV infection in Taiwan are similar to those in Western Countries in recent years although HPV vaccination is not prevalent yet [21]. In fact, our findings revealed that the percentage of HPV positive OPC in Northern Taiwan (33%), where it is more urbanized with higher prevalence of HPV infection and lower prevalence of betel nut chewing [16, 22], is higher than that in other regions (22.5%) and rose from 25 to 38% during the period of 1999–2014. This trend is similar to another single hospital-based cohort in Northern Taiwan [12]. This suggests that the percentage of HPV positive OPC will rise and surpass HPV negative OPC, becoming a common head and neck cancer in the near future.

Our findings showed that HPV 16 (70%) is the most common subtype, similar to other countries. What is different is that HPV 58 (12%) is the second common subtype and only 1% of all HPV is subtype 18 in Taiwan [1–5]. This pattern is similar to the studies on HPV in cervical cancer [18] and another study on OPC [12] in Taiwan. HPV16 and HPV58 were found to cause a higher risk of cervical cancer than other carcinogenic HPV types but the etiology remains unclear [18]. Nowadays, there are three HPV vaccines available in Taiwan for protecting young girls against cervical cancer: AS04-HPV-16/18v (Cervarix, GSK, Belgium) against HPV16/18, 4vHPVv (Gardasil, Merck, USA) against HPV6/11/16/18, and 9vHPVv (Gardasil 9, Merck, USA) against HPV6/11/16/18/31/33/45/52/58 [23]. 9vHPVv may be considered as an better option for both female cervical cancer and male/female HPV positive OPC prevention in Taiwan given it’s protection against HPV 16/18/58.

In Taiwan, most betel nut chewers are male, and female seldom chews betel nut or smoke cigarette [16]. The majority of OPC patients without ABC exposure was HPV positive in the current study. These can explain why most male OPC patients are HPV negative while most female OPC patients are HPV positive in Taiwan. Similar to the studies in countries where betel nut chewing is rare [1–5], HPV positive OPC in Taiwan had smaller primary tumor and more advanced neck disease, and nearly half of all tonsillar cancers were HPV positive but the majority of tongue base and soft palate cancers were HPV negative.

The current study showed that p16 staining is still a good surrogate indicator for the presence of HPV infection in OPC demonstrating that p16 staining is also helpful in finding primary tumor origin for neck metastasis of unknown origin in Taiwan according to NCCN guideline [24]. Our findings also showed that the presence of HPV infection by PCR or positive p16 staining in primary OPC tumor can be an indicator of prognosis after treatment with chemoradiation. Similar to Western countries [7] but different than another study in Taiwan [12], the current study showed that smoking is significantly worsens the prognosis of HPV positive OPC patients. It is difficult to separately evaluate the effect of betel nut chewing on survival of HPV positive OPC because almost all betel nut chewers are also smokers. As class I carcinogen [25], betel nut causes heavy mutation burdens in head and neck cancers [6] and probably worsens the prognosis of HPV positive OPC. Therefore, this study considered betel nut chewing or/and cigarette smoking as one factor because of practical reason and showed that patients with HPV positive OPC but no betel nut/cigarette exposure had the best treatment outcome but patients with HPV positive OPC and betel nut/cigarette exposure had significantly worse treatment outcome. Patients with HPV negative OPC and betel nut/cigarette exposure had the worst outcome. In contrast, we did not find alcohol drinking was associated with poorer prognosis of HPV positive OPC [12]. An explanation for this difference may be due to difference in the definition of alcohol exposure and poor reliability of self reported alcohol drinking.

## Conclusion

The incidence of HPV positive OPC is increasing along with HPV negative OPC in the past decades, which leads to stably low percentage of HPV positive OPC in Taiwan. HPV positive OPC may become an important head and neck cancer when the incidence of HPV negative OPC starts to decline in the near future. P16 is a useful surrogate marker for HPV infection in OPC and a good prognostic indicator for treatment outcome of OPC. Patients with HPV positive OPC but no betel nut/cigarette exposure yield excellent prognosis after treatment. Betel nut/cigarette exposure significantly worsens the prognosis of HPV positive OPC.

## Data Availability

The datasets used and/or analyzed during the current study are available from the corresponding author on reasonable request.
